# Neurophysiological and cognitive enhancements in autonomous sensory meridian response identified using heart rate variability and electroencephalography connectivity

**DOI:** 10.3389/fpsyg.2025.1652185

**Published:** 2025-11-24

**Authors:** In-Nea Wang, Hayom Kim, Hakseung Kim, Ho-Jin Yoon, Jun-Su Park, Jung Bin Kim, Dong-Joo Kim

**Affiliations:** 1 Department of Brain and Cognitive Engineering, Korea University, Seoul, Republic of Korea; 2 Department of Neurology, Korea University Anam Hospital, Korea University College of Medicine, Seoul, Republic of Korea; 3 Department of Convergence Medicine, Asan Medical Center, University of Ulsan College of Medicine, Seoul, Republic of Korea; 4 NeuroTx, Co., Ltd., Seoul, Republic of Korea

**Keywords:** autonomous sensory meridian response, heart rate variability, cognition, electroencephalography, electrocardiography, autonomic nervous system

## Abstract

**Introduction:**

Autonomous sensory meridian response (ASMR) is a sensory-emotional phenomenon characterized by tingling sensations and relaxation, typically elicited by auditory or visual stimuli. Although anecdotal reports suggest potential cognitive and physiological benefits, empirical evidence remains limited. Furthermore, objective physiological monitoring of ASMR-related responses has yet to be systematically explored.

**Methods:**

Twenty healthy Korean adults underwent 5-min exposure to natural sound-based ASMR stimuli. Cognitive assessments, including the Trail Making Test Part B and a delayed word recall task, were administered before and after stimulation. Simultaneously, electroencephalography (EEG) and electrocardiography were recorded to examine power spectral density (PSD), functional connectivity (FC), and HRV indices. Gaussian Mixture Modeling (GMM) was applied to HRV features to classify responders.

**Results:**

Post-ASMR stimulation, participants demonstrated significant cognitive improvements (Trail Making Test: *P* = 0.004; Delayed Recall: *P* = 0.022). EEG analyses revealed increased PSD in frontoparietal regions and enhanced FC, particularly in beta and gamma bands. HRV measures showed elevated parasympathetic activity. GMM clustering identified two groups; responders exhibited greater cognitive enhancement and FC changes than non-responders. Correlation analyses showed positive associations between HRV indices and memory performance, and a negative correlation between beta-band prefrontal FC and recall.

**Discussion:**

Auditory ASMR stimulation improves executive and memory functions, potentially through modulating neural connectivity and autonomic function. HRV indices serve as practical, non-invasive markers for real-time monitoring of auditory-induced neurocognitive changes, offering potential for personalized cognitive enhancement strategies.

## 1 Introduction

Human beings have long been fascinated by the intricate interplay between sensory stimuli and the mind. Across various cultures and historical contexts, individuals have employed visual, auditory, and tactile techniques to induce relaxation, improve mood, or enhance concentration (Poerio et al., 2018; Smith et al., 2019). Practices such as meditation, music therapy, and environmental enrichment exemplify how sensory interventions modulate mental states through external cues (Kovacevich and Huron, 2018; Swart, 2023). These interventions reflect an interdisciplinary convergence among psychology, neuroscience, physiology, and media studies, wherein researchers aim to characterize novel phenomena that bridge sensory input with physical responses (Engelbregt et al., 2022; Battaglia et al., 2023; Battaglia et al., 2024; Di Gregorio et al., 2024; Gregorio and Battaglia, 2024).

One phenomenon that has garnered significant scientific attention and public interest is autonomous sensory meridian response (ASMR). ASMR is often described as a pleasant, tingling sensation originating at the scalp and cascading down the neck, arms, and spine (Smith et al., 2017; Roberts et al., 2021; Engelbregt et al., 2022). This tingling can be elicited through triggers such as soft voices, gentle tapping sounds, slow hand movements, and natural sounds like rustling leaves (Smith et al., 2017; Roberts et al., 2021). Although somatic, ASMR often induces feelings of calm, relaxation, and mild euphoria (Roberts et al., 2021; Engelbregt et al., 2022). Over the last decade, ASMR has transitioned from a niche, anecdotal experience into a recognized topic of scientific inquiry, aided by the rise of ASMR-related content on platforms like YouTube (Engelbregt et al., 2022).

The physiological and psychological effects of ASMR have been explored using neuroimaging techniques such as electroencephalography (EEG) and functional magnetic resonance imaging (fMRI). EEG, with its high temporal resolution, enables real-time monitoring of neurophysiological changes during ASMR and has revealed increased power spectral density (PSD) in frontal and parietal regions associated with cognitive and sensory processing (Swart et al., 2022a; Yu et al., 2023; Seifzadeh and Kostek, 2024). However, EEG primarily captures cortical activity, whereas fMRI, with its superior spatial resolution, highlights activation in subcortical regions such as the hippocampus, amygdala, and thalamus—areas involved in sensation, attention, and emotional regulation (Attal and Schwartz, 2013). Studies comparing ASMR responders to controls demonstrate heightened activity in the right cingulate gyrus, paracentral lobule, and bilateral thalamus, supporting the influence of ASMR on cognitive and emotional processes (Smith et al., 2019).

The potential relationship between ASMR and the autonomic nervous system (ANS) is another area of interest. The ANS regulates physiological balance between sympathetic (fight-or-flight) and parasympathetic (rest-and-digest) activities (Forte et al., 2019; Di Gregorio et al., 2024). Heart rate variability (HRV), a well-established measure of autonomic function, reflects this balance and has been widely studied as an indicator of adaptive physiological regulation (Forte et al., 2019; Battaglia et al., 2023; Battaglia et al., 2024; Gregorio and Battaglia, 2024). Higher HRV has been associated with cognitive performance, emotional wellbeing, and stress resilience (Kim et al., 2006; Hansen et al., 2009). Preliminary evidence suggests that ASMR may promote parasympathetic activation, as individuals often report feelings of calm and relaxation during ASMR experiences (Kovacevich and Huron, 2018; Mahady et al., 2023). Reductions in heart rate and other physiological markers support this hypothesis, yet the broader influence of ASMR on HRV and its implications for cognitive performance remain underexplored.

Cognitive function encompasses attention, executive function, and memory, which are crucial for daily activities and wellbeing. Identifying whether ASMR reliably alters these dimensions is essential, as it may offer non-invasive methods for enhancing mental performance (Swart, 2023). Studies link ASMR exposure to improved executive function and memory recall (Del Campo, 2019; Kim et al., 2024), potentially due to enhanced functional connectivity (FC) in brain networks involved in attention and memory. Increased FC in the default mode network (DMN), particularly between the posterior cingulate cortex, superior temporal gyri, and lingual gyrus, has been observed during ASMR experiences (Lee et al., 2020). These findings suggest ASMR engages neural pathways critical for cognitive and emotional regulation. Additionally, regions like the medial prefrontal cortex (mPFC) and ventromedial prefrontal cortex (vmPFC)—key for autonomic and emotional regulation—highlight the interplay between central and autonomic processes during ASMR (Etkin et al., 2011; Patron et al., 2019).

Although previous studies have reported various psychological and physiological effects of ASMR, most evidence has relied heavily on self-report questionnaires to assess ASMR responsiveness, such as the ASMR-15, ASMR Experience Questionnaire (AEQ), or ASMR Trigger Checklist (ATC) (Roberts et al., 2019; Swart et al., 2022b; Poerio et al., 2023). While these tools provide valuable subjective perspectives, they are inherently limited by individual interpretation, recall bias, and contextual variability (Morales et al., 2021). Such dependence on subjective reporting makes it difficult to establish consistent and reproducible criteria for quantifying ASMR experiences or their physiological correlates. Consequently, identifying objective physiological indicators that can complement or substitute for self-reports is essential for advancing ASMR research. Heart rate variability (HRV) represents a promising candidate, as it provides a quantifiable, reproducible index of autonomic regulation that can be continuously monitored in real time (Farmer et al., 2014). Emerging evidence suggests HRV indices effectively capture differences in autonomic and cognitive responses to sensory stimuli, including music and relaxation techniques (Hansen et al., 2009; Forte et al., 2019; Morales et al., 2021). Given the association between ASMR and parasympathetic activation, HRV may serve as an objective marker for distinguishing responders from non-responders. By analyzing HRV alongside cognitive and neurophysiological measures, researchers can uncover mechanisms that differentiate these groups and provide insights into the central–autonomic interplay during ASMR exposure (Hansen et al., 2009).

Building upon this foundation, the present study aimed to evaluate whether HRV parameters—non-invasive, easily measurable indices obtainable through wearable devices—can serve as real-time monitoring indicators of ASMR-induced changes in cognitive performance, brain network activity, and autonomic function. The hypotheses of this study are summarized in Table 1. To this end, HRV-based measures were compared with EEG-derived FC indices, enabling direct comparison between peripheral autonomic markers and central neural activity. Furthermore, Gaussian Mixture Model (GMM) clustering was applied using HRV change features to identify distinct physiological response patterns. This exploratory analysis was designed to assess whether HRV-based clustering could differentiate participants exhibiting ASMR-related improvements in cognition and autonomic regulation from those showing minimal responses. Through this integrative approach combining HRV monitoring, EEG network analysis, and cognitive assessment, this study sought to establish a foundation for wearable-based real-time monitoring of ASMR-related neurophysiological modulation.

**TABLE 1:** Summary of hypotheses tested in this study.

Hypothesis	Description
H1	Exposure to ASMR stimuli enhances cognitive performance.
H2	ASMR increases HRV, indicating enhanced parasympathetic activity.
H3	Individuals identified as ASMR responders show greater cognitive and physiological changes than non-responders.
H4	HRV indices reflect differential autonomic and cognitive responses to ASMR, serving as potential physiological markers for monitoring auditory-induced changes.

## 2 Materials and methods

### 2.1 Participants and cognitive assessment

The study included 20 participants (10 women, 10 men; mean age, 26.050 ± 2.188 years), all of whom were affiliated with Korea University and Korea University Medical Center in South Korea. Participants were homogenous in terms of race and ethnicity, as all were East Asian and specifically Korean. In addition, participants had a high level of education, as they were graduate students or research staff affiliated with the aforementioned institutions. Participants had normal auditory function, either normal or corrected-to-normal vision, and no neurological or psychiatric issues. All participants underwent a neurological examination and detailed interview to ensure the following inclusion criteria were met: (a) no neurological abnormalities and no global cognitive impairment with Mini-Mental State Examination (MMSE) ≥ 28/30 (Ciesielska et al., 2016); (b) no history of neurological, psychiatric, or systemic disorders; and (c) no history of alcohol or drug abuse prior to the study. The menstrual cycle phase in female participants was not controlled, which may have contributed to variability in autonomic measures. Written informed consent was obtained from all participants before the study. This research was conducted in accordance with the tenets of the Declaration of Helsinki, and the experimental protocol was approved by the Ethics Committee of Korea University Anam Hospital (No. 2022AN0246).

The overall neurocognitive function was assessed using the Korean version of the MMSE. The evaluated domains and tests were as follows (Park and Kwon, 1990):

(i) Attention and working memory were assessed using the Digit Span Forward (participants recalled up to nine digits in sequence) and Digit Span Backward tests (participants recalled up to eight digits in reverse order), along with a Target Detection Task requiring responses to tapping. (ii) Executive function was evaluated using a modified version of the Trail Making Test (TMT) Part B, in which participants connected eight digits and five Korean letters sequentially. Each participant completed two equivalent but non-identical test sets—one before and one after auditory stimulation—to control for repetition or learning effects. (iii) Memory was assessed using a short-term memory recall task involving two learning trials with five words, followed by a delayed recall task conducted after 5 min. To minimize practice effects, different but comparable word sets were used between pre- and post-stimulation sessions.

### 2.2 Experimental procedure

The experimental procedure consisted of three stages: pre-ASMR, during ASMR, and post-ASMR. EEG and ECG recordings, as well as psychological assessments, were conducted during the pre-ASMR and post-ASMR stages, while auditory stimulation occurred during the ASMR stage.

During EEG and ECG recordings, participants lay comfortably in a supine position with their eyes closed in a quiet, isolated room to minimize muscle activity and eye movement artifacts. This posture ensured stable physiological measurements and a relaxed resting state conducive to accurate signal acquisition.

For the cognitive assessments, participants were asked to sit upright with their eyes open to complete the tasks. This posture change was applied only during the cognitive testing periods to prevent postural or visual factors from confounding the electrophysiological recordings.

The auditory stimulation during the ASMR stage consisted of a 5-min segment of natural sounds—including wind, rustling leaves, and bird chirping—selected based on prior literature and popular ASMR content known to elicit relaxation and tingling sensations (Sakurai et al., 2023). The stimuli were delivered at a constant volume through the built-in speakers of an iPad (Apple, Cupertino, California, United States).

Throughout the experiment, participants were assigned unique IDs to maintain anonymity. Researchers monitored participants in real time via an internal camera to ensure adherence to protocol without physical interference. Each stage was time-stamped to allow precise pre–post comparisons of EEG, ECG, and behavioral parameters.

### 2.3 Data acquisition and preprocessing

EEG and ECG signals were collected for 3 min during both the pre-ASMR and post-ASMR stages. EEG data were acquired from 19 scalp electrodes (Fp1, F7, T7, P7, O1, Fp2, F8, T8, P8, O2, F3, C3, P3, F4, C4, P4, Fz, Cz, Pz) in accordance with the international 10–20 system, using a 32-channel recording device (Xltek^®^ EEG Brain Monitor; Natus Neurology, Oakville, Ontario, Canada) at a sampling rate of 512 Hz. ECG signals, heart rate, and RR intervals were measured using a commercial wearable device (MAXREFDES103#, Maxim Inc.) at a sampling rate of 19 Hz. Data collection was monitored in real-time by two specialized neurologists from a separate room.

Initial analysis involved partitioning of the EEG and ECG signal data into segments, synchronized with the start and end times of each phase of the experiment, using MATLAB (MathWorks, Natick, MA). Within the 3-min pre-ASMR and post-ASMR recordings, 2 min of data were selected by excluding the first and last 30 s of each section. Subsequently, preprocessing was performed for each signal. EEG was preprocessed in three stages using the MNE-Python software package (Gramfort et al., 2013): (i) extraction of frequency ranges from 1 to 70 Hz using a FIR bandpass filter; (ii) random selection of 10 non-continuous 2-s epochs for FC analysis; (iii) and segregation of signal into delta (0.5–4 Hz), theta (4–8 Hz), alpha (8–13 Hz), beta (13–30 Hz), and gamma (30–50 Hz) frequency ranges using a Welch bandpass filter (Welch, 1967). EEG coherence was calculated using 10 non-consecutive epochs, each lasting 2 s, to capture transient neural synchronization following auditory stimulation. This relatively short epoch duration was deliberately chosen to align with the study’s real-time monitoring framework, in which EEG and ECG signals were synchronously recorded to evaluate whether auditory stimuli could elicit immediate neurophysiological and autonomic changes. Because ASMR responses are often short-lived and vary in duration across individuals, non-consecutive short segments were analyzed to reflect the temporal dynamics of transient ASMR-related activity while minimizing the influence of non-stationary artifacts. Averaging coherence values across multiple short, non-overlapping epochs improved estimate reliability while preserving sensitivity to rapid physiological fluctuations. For ECG, preprocessing was performed in five steps using Kubios HRV Standard software version 3.5 (Tarvainen et al., 2014): (i) adaptive QRS detection and pulse wave detection for RR interval calculation; (ii) identification of noise segments that could distort analysis; (iii) automatic artifact correction using available beat correction algorithms in the Kubios HRV standard; (iv) removal of abnormal trends in the inter-beat interval time series; and (v) transformation of the signals into the frequency domain using Fast Fourier Transform. The frequency domain was further divided into very low frequency (VLF, 0–0.04 Hz), low frequency (LF, 0.04–0.15 Hz), and high frequency (HF, 0.15–0.4 Hz) (Mali et al., 2014).

### 2.4 Feature extraction and Gaussian Mixture model clustering

After preprocessing for each signal, relative PSD (Solomon, 1991) and coherence-based FC (Thatcher et al., 1986) were extracted from the EEG signals using the MNE-Python library, whereas HRV parameters were extracted from the ECG signals using Kubios HRV Standard software (Tarvainen et al., 2014). The relative PSD was normalized according to the frequency bands for each channel, resulting in 95 PSD features (obtained by multiplying the 5 frequency bands with 19 channels), represented in the format of “frequency-channel.” Similarly, coherence calculations displayed connectivity between channels for each frequency band, yielding a total of 1,805 FC features. By considering undirected connectivity and excluding redundant connections between the same channels, 855 FC features were extracted and represented in the format of “frequency channel-channel.”

For each participant, difference scores were calculated for all cognitive, HRV, and FC measures by subtracting pre-ASMR values from post-ASMR values. This approach ensured that clustering was based on relative changes attributable to ASMR stimulation, minimizing the influence of baseline variability (Norman et al., 2014). A GMM, an unsupervised learning method widely used for handling outliers and clustering data, was employed to determine whether participants could be differentiated based on heart rate variability (HRV) indicators (Lam et al., 2015). The model estimates the probability of each data point belonging to a specific Gaussian cluster using the Expectation–Maximization algorithm, which iteratively optimizes parameter estimation while being less constrained by initial parameter settings (McLachlan and Rathnayake, 2014). GMM clustering was implemented using Scikit-learn, a Python-based machine learning library (Pedregosa et al., 2011). Following clustering, outliers classified as -1 were excluded, resulting in the removal of two participants. To complement the absence of subjective data, a five-fold cross-validation and silhouette analysis were performed to evaluate the robustness and internal consistency of the clustering results derived from HRV features.

### 2.5 Statistics

To assess neurophysiological and autonomic changes between the pre- and post-ASMR conditions, non-parametric paired tests were applied because the data did not meet normality assumptions. Specifically, the Wilcoxon signed-rank test was performed using the *scipy.stats* module in Python to compare pre- and post-ASMR differences within participants for EEG, ECG-derived HRV, and behavioral indices.

For correlation analyses, Spearman’s rank correlation was conducted to examine associations among cognitive performance, EEG metrics (PSD and FC), and HRV parameters. In these analyses, the differences between post-ASMR and pre-ASMR values were used to capture stimulation-induced changes.

To evaluate intergroup differences between participants classified as responders and non-responders based on the GMM clustering of HRV features, independent-samples Mann–Whitney *U*-tests were conducted using IBM SPSS Statistics for Windows, version 27.0 (IBM Corp., Armonk, NY, United States).

For EEG analyses, multiple comparisons across frequency bands and electrode sites were corrected using the false discovery rate (FDR) correction (corrected *P* < 0.05) to control for type I error inflation. Statistical significance for all analyses was set at *P* < 0.05 (two-tailed).

## 3 Results

All participants demonstrated normal cognitive function, with mean MMSE scores within the normal range (29.36 ± 0.74 for responders and 29.75 ± 0.50 for non-responders, *P* = 0.442). No significant group differences were observed in age (26.07 ± 2.30 vs. 25.25 ± 1.89 years, *P* = 0.442) or sex distribution (57.1 vs. 50.0% female, *P* = 1.000) (Table 2).

**TABLE 2:** Demographic and baseline characteristics of responders and non-responders.

Variables	Responder (*N* = 14)	Non-responder (*N* = 4)	*P-* value
Sex (women, %)	57.14	50.000	1.000^a^
Age (years)	26.07 ± 2.30	25.25 ± 1.89	0.442^b^
MMSE score	29.36 ± 0.74	29.75 ± 0.50	0.442^b^

Values for age and MMSE score were presented as mean ± standard deviation. Sex was reported as the percentage of women in each group.

*^a^*The *P*-value for sex was calculated using Fisher’s exact test.

*^b^*The p *P*-values for age and MMSE score were calculated using the Mann-Whitney *U*-test. MMSE, Mini-Mental State Exam.

### 3.1 Cognitive outcomes

**FIGURE 1:**
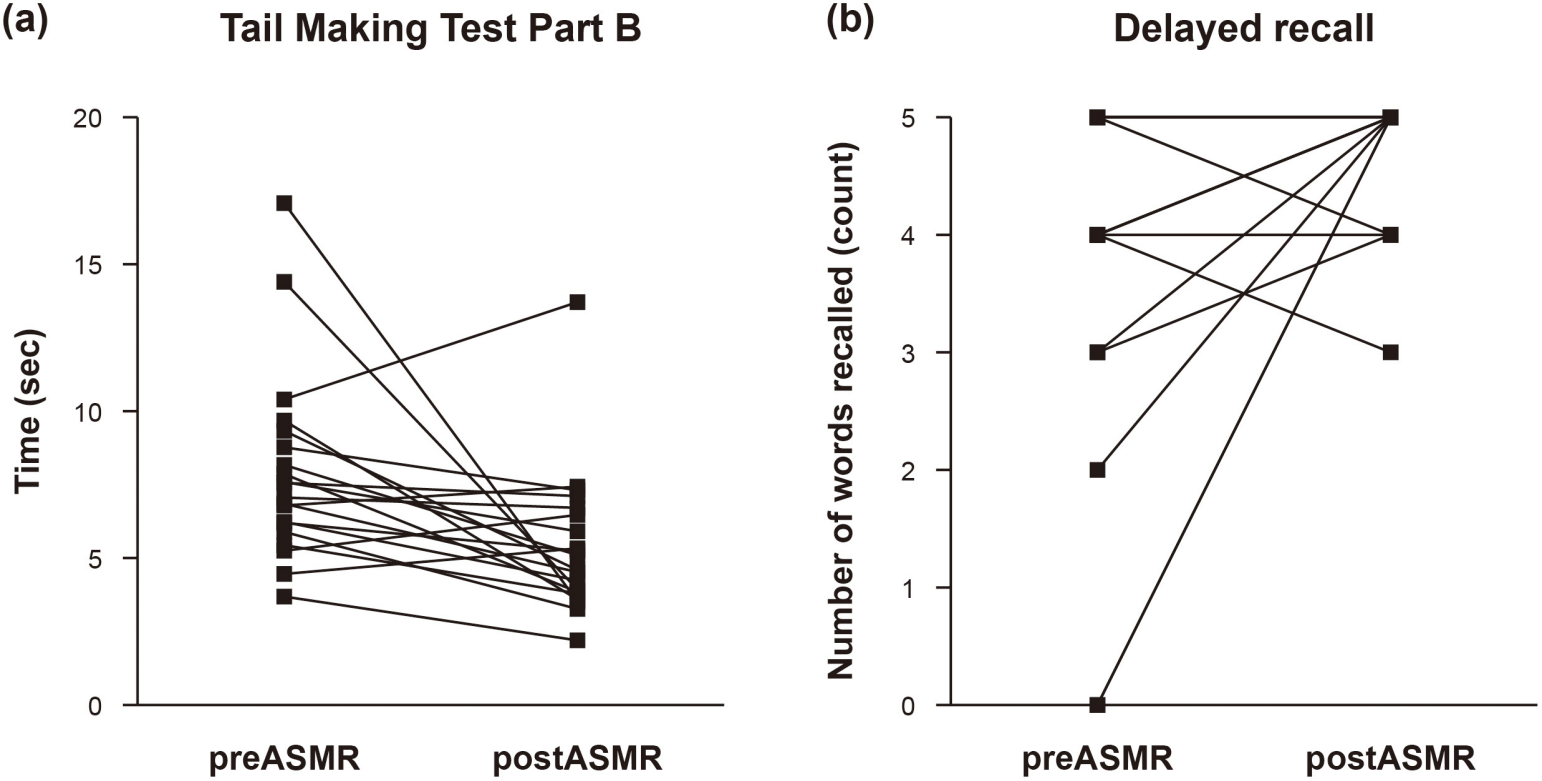
Changes in cognitive function after autonomous sensory meridian response. Impact of autonomous sensory meridian response (ASMR) on executive function was evaluated by examining individual changes in **(a)** the completion time of Trail Making Test Part B and **(b)** the number of words recalled from memory.

Behavioral analysis revealed significant post-ASMR improvements in executive function and memory. The mean completion time on the Trail Making Test Part B decreased from 7.93 ± 3.20 s at baseline to 5.39 ± 2.46 s post-stimulation (*P* = 0.004), indicating enhanced cognitive flexibility and task-switching ability. Similarly, performance on the delayed recall task increased from 3.85 ± 1.56 to 4.75 ± 0.55 words recalled (*P* = 0.022), suggesting improved memory retention (Table 3). Figure 1 visualizes individual-level improvements in both TMT-B completion times and recall accuracy, illustrating consistent cognitive enhancement across participants.

**TABLE 3:** Comparison of cognitive performance between pre-ASMR and post-ASMR stages.

Cognitive measurements	Pre-ASMR	Post-ASMR	*P*-value*
Trail making test part B (completion times, seconds)	7.93 ± 3.20	5.39 ± 2.46	0.004
Delayed recall (number of recalled words, count)	3.85 ± 1.56	4.75 ± 0.55	0.022

Values were presented as mean ± standard deviation.

*The *P-*values were calculated using the Wilcoxon signed-rank test.

### 3.2 EEG power spectral density

**FIGURE 2:**
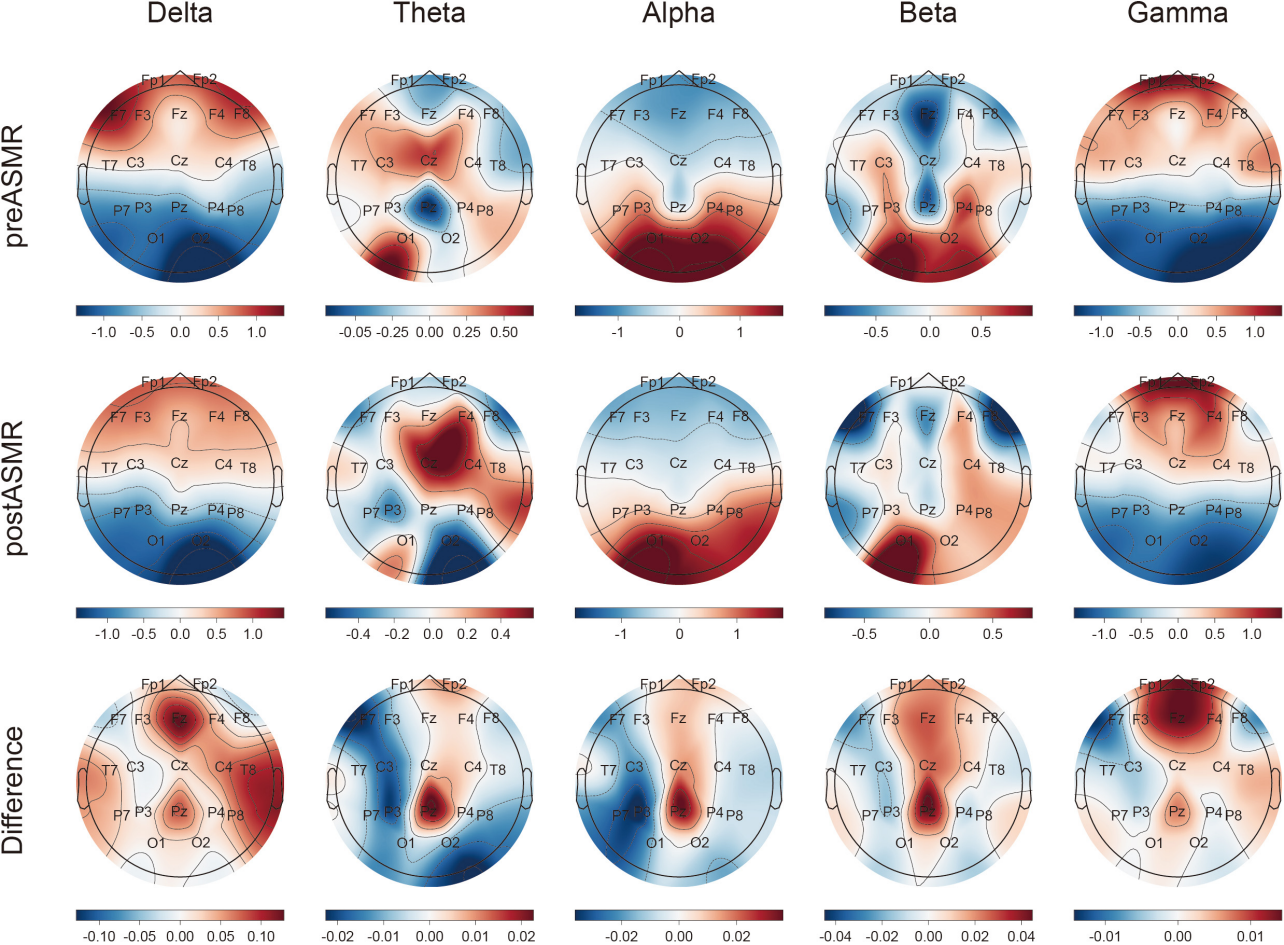
Topographical map of relative power spectral density by each frequency band. Topographical plots are shown representing pre-ASMR and post-ASMR resting state for all participants (upper and middle panels, respectively). The lower panel depicts differences in related power spectral density (PSD) values between post-ASMR and pre-ASMR stages for each channel and band and its normalization.

Topographic analysis demonstrated spatially localized increases in relative PSD after ASMR exposure. In particular, delta-band power increased in temporal regions, while beta- and gamma-band power rose in the frontal lobe. The fronto-parietal electrodes (especially Fz and Pz) exhibited marked enhancement across multiple frequency bands (Figure 2). Statistically significant PSD increases were confirmed in Supplementary Table 1.

### 3.3 Functional connectivity

**FIGURE 3:**
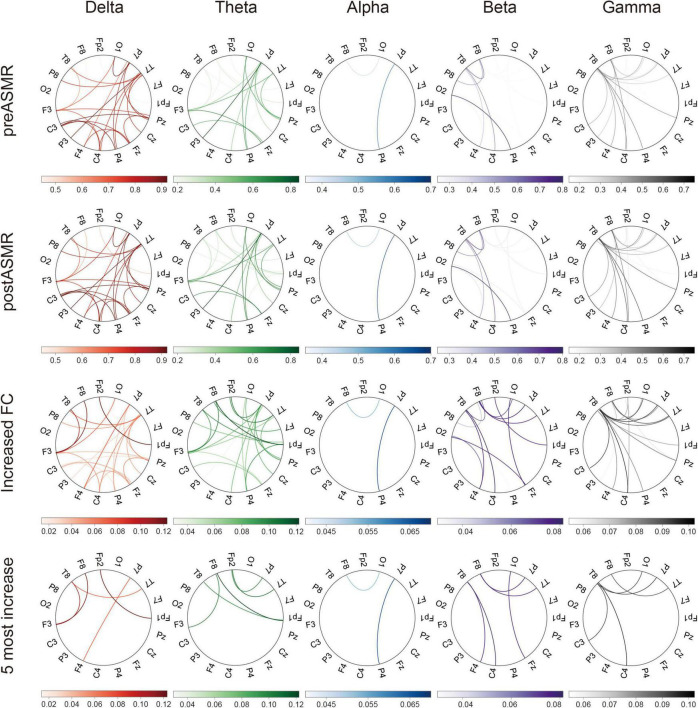
Functional connectivity (FC) in terms of coherence. The circular plots show the coherence between 19 pairs of scalp electroencephalography electrodes in each frequency. Each column corresponds to a frequency band, with the upper and lower panels showing coherence circular plots for resting-state EEG data in the pre-ASMR and post-ASMR stages, respectively. The lower panel shows the difference in coherence values, obtained by subtracting the pre-ASMR from the post-ASMR, while the last row displays the top five lines with the greatest increase in difference coherence in each frequency band. Only coherence values with FDR-corrected *P* < 0.05 are displayed. ASMR, autonomous sensory meridian response; EEG, electroencephalography.

EEG coherence analysis revealed significantly strengthened FC across all frequency bands post-ASMR (Figure 3). The right temporal (T8) and right frontal (F8) electrodes emerged as principal hub regions, suggesting enhanced inter-regional synchronization. Supplementary Table 2 details the specific frequency-band connections showing significant increases, particularly in beta and gamma bands.

### 3.4 Correlation analyses

**FIGURE 4:**
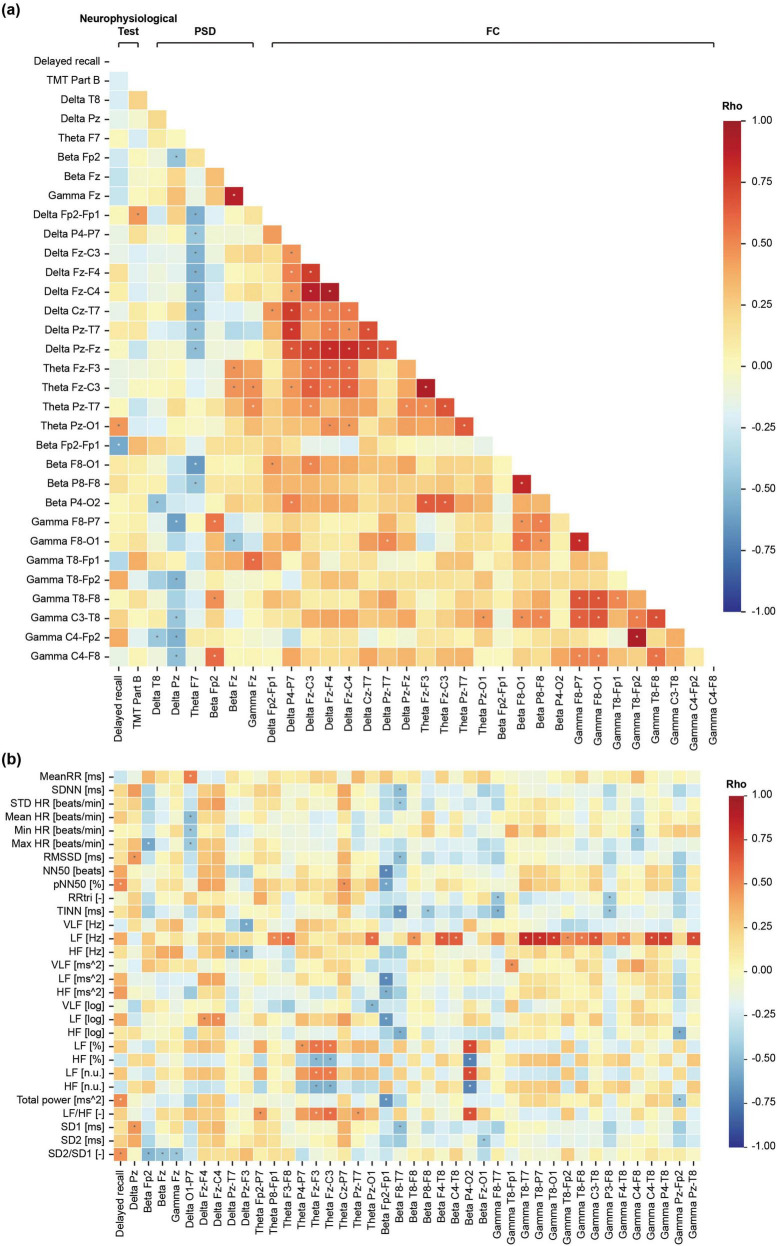
Spearman correlation coefficient matrix between features with **(a)** significant differences between pre-ASMR and post-ASMR stages and **(b)** heart rate variability (HRV) features. ASMR, autonomous sensory meridian response; PSD, power spectral density; FC, functional connectivity.

**FIGURE 5:**
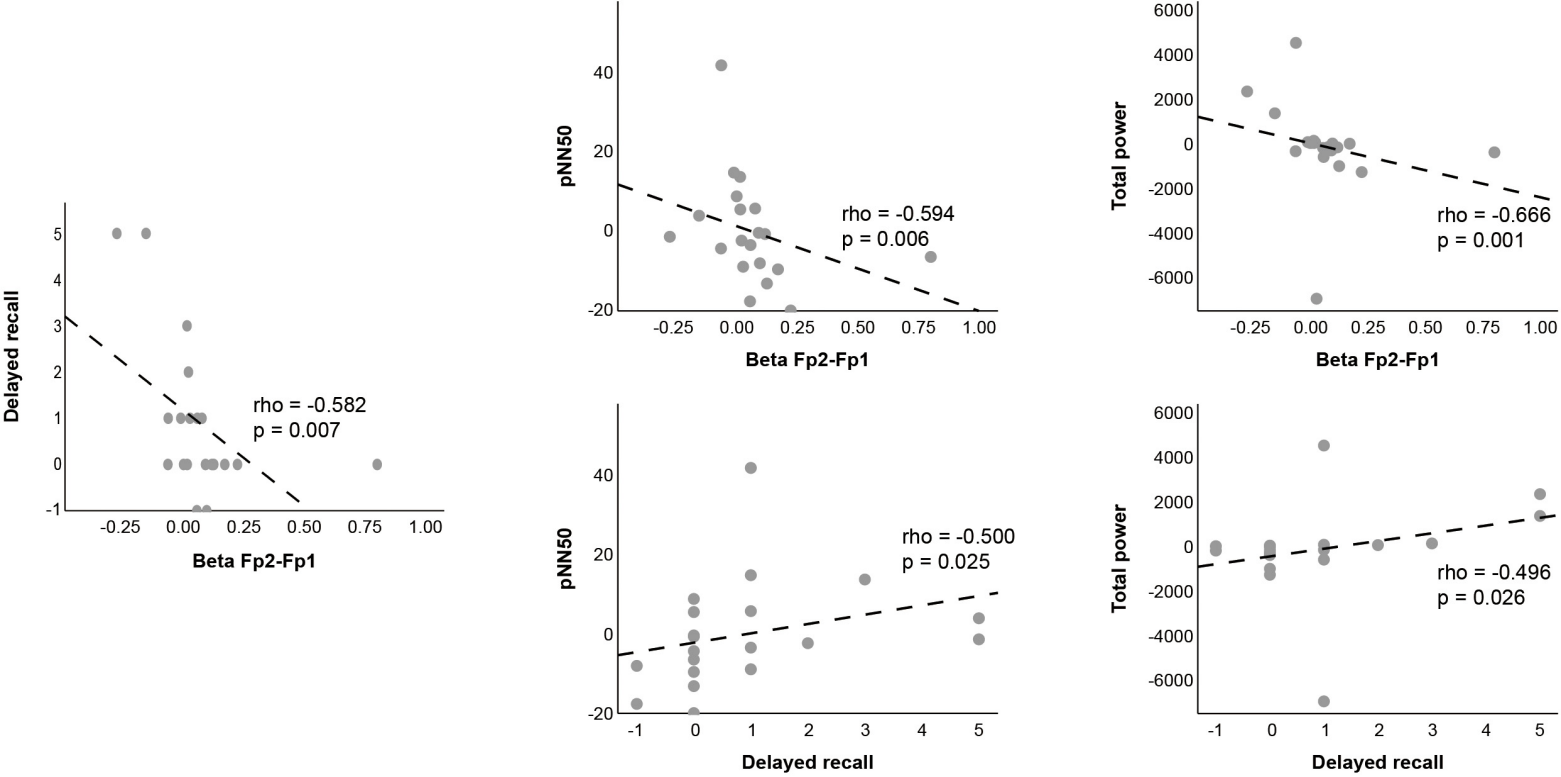
Scatter plots of delayed recall, beta Fp2-Fp1, pNN50, and total power.

Correlational analyses (Figures 4, 5) demonstrated a significant negative relationship between beta-band prefrontal connectivity (Fp2–Fp1) and both delayed recall and HRV indices, suggesting that lower prefrontal coupling facilitates better memory retrieval. Conversely, HRV indices—including pNN50 and total power—showed positive correlations with delayed recall performance (Supplementary Table 3), indicating that increased parasympathetic modulation accompanies improved memory outcomes.

### 3.5 HRV-based clustering

GMM clustering based on HRV parameters (pNN50 and total power) yielded two clusters (Cluster 1, *n* = 14; Cluster 2, *n* = 4). Cluster 1—designated as “responders”—showed greater improvements in both cognition and EEG indices. Specifically, responders demonstrated larger gains in delayed recall (1.07 ± 1.44 vs. –0.50 ± 0.58; *U* = 6.00, *Z* = –2.47, *P* = 0.018) and significantly increased FC in multiple bands, including theta P3–P7 (*P* = 0.025), theta Pz–P7 (*P* = 0.018), theta Pz–O1 (*P* = 0.012), beta F8–T7 (*P* = 0.035), and gamma T8–T7 (*P* = 0.035) (Table 4; Supplementary Table 4). Five-fold cross-validation of the GMM clustering showed good internal stability, with an average adjusted Rand index of 0.71 ± 0.08 and a mean silhouette coefficient of 0.63 ± 0.05. These values indicate consistent participant classification and acceptable intra-cluster cohesion despite the small and unbalanced sample. Although self-report confirmation of ASMR was unavailable, HRV-based clustering revealed coherent physiological groupings, suggesting that simple autonomic indices may differentiate individual response profiles to auditory stimuli.

**TABLE 4:** Mann–Whitney *U*-test results for the cluster using heart rate variability (HRV) features.

Features	GMM clustering		Mann-whitney *U*-test		
	Cluster 1 (*N* = 14)	Cluster 2 (*N* = 4)	U	Z	*P-*value
Delayed recall	1.07 ± 1.44	–0.50 ± 0.58	6.00	–2.47	0.018
Theta P3-P7	0.05 ± 0.07	–0.01 ± 0.03	7.00	–2.23	0.025
Theta Pz-P7	0.12 ± 0.20	–0.05 ± 0.06	6.00	–2.34	0.018
Theta Pz-O1	0.12 ± 0.21	–0.07 ± 0.08	5.00	–2.44	0.012
Beta F8-T7	0.11 ± 0.21	–0.03 ± 0.06	8.00	–2.12	0.035
Gamma T8-T7	0.13 ± 0.21	–0.03 ± 0.04	8.00	–2.12	0.035

Values are indicated mean ± standard deviation of difference (post-ASMR—pre-ASMR).

### 3.6 Summary

Collectively, ASMR auditory stimulation produced statistically significant improvements in executive and memory performance, accompanied by increased cortical activation and functional connectivity, as well as elevated HRV indices reflecting parasympathetic engagement. The combination of EEG and HRV findings supports HRV features as potential biomarkers for identifying ASMR responders and highlights the central–autonomic coupling underlying ASMR-induced cognitive enhancement.

## 4 Discussion

This study investigated the effects of ASMR auditory stimulation on cognitive function and neurophysiological changes, providing novel insights into its mechanisms and potential therapeutic applications. By integrating cognitive assessments, neurophysiological markers, and HRV indices, the findings demonstrated significant improvements in executive function and memory recall. Furthermore, HRV features were identified as objective biomarkers for distinguishing responders from non-responders, supporting the feasibility of personalized interventions. This discussion contextualizes the findings within the broader literature, explores their implications, and addresses challenges and directions for future research.

The results revealed notable cognitive enhancements following auditory stimulation. Reduced completion times for the Trail Making Test Part B and improved delayed recall suggest improved cognitive flexibility, task-switching, and long-term memory retention (Liston et al., 2006; Tsoi et al., 2017), functions closely associated with the prefrontal cortex and the fronto-parietal network (Arbuthnott and Frank, 2000). Increased beta band activity observed in frontal regions further corroborates these findings, as this frequency band is implicated in working memory and task-switching capabilities (Ren et al., 2012). Enhancements in delayed recall likely reflect hippocampal activation, as evidenced by increased delta band activity in the temporal lobe, a region critical for memory encoding and retrieval (Harmony, 2013). EEG analyses emphasized the neurophysiological underpinnings of these cognitive effects, with increased PSD across all frequency bands in the Fz and Pz regions highlighting augmented neural activity in areas associated with executive function and memory (Liu et al., 2023). Additionally, enhanced FC in T8 and F8 regions underscores the strengthening of neural interactions, likely contributing to sensory-cognitive integration and emotional regulation (Seifzadeh and Kostek, 2024). Compared to previously reported EEG responses to general emotional sound stimuli, such as music or nature sounds, which are typically characterized by increased alpha and theta power linked to relaxation and emotional engagement, the observed patterns in this study—including increased beta power and enhanced FC in regions associated with executive function and memory—suggest that ASMR activates unique neurophysiological mechanisms (Seifzadeh and Kostek, 2024). These distinctions imply that ASMR stimulation elicits specific effects on cognitive processes beyond the general arousal or relaxation effects typically associated with emotional sounds, supporting its potential as a targeted intervention for enhancing cognitive performance.

Correlation analysis revealed significant relationships among HRV indices, FC, and cognitive outcomes. Among the HRV indicators, pNN50 reflects parasympathetic nervous system activity (Hanslmayr et al., 2011), and the total power represents overall autonomic nervous system activity (Koenig et al., 2014). Reduced beta band connectivity between Fp1 and Fp2 was negatively correlated with delayed recall, indicating that lower connectivity in this frequency band may facilitate memory retrieval. Simultaneously, positive correlations between HRV indices (pNN50 and total power) and delayed recall emphasize the role of parasympathetic activation in optimizing conditions for cognitive performance (Symanski et al., 2022). These findings provide a comprehensive understanding of the interplay between central and autonomic nervous system regulation during stimulation. The identification of HRV indices as potential markers for responsiveness to stimulation represents a significant advancement. These biomarkers allow the assessment of individual variability in neural and cognitive responses, enabling tailored approaches to maximize therapeutic outcomes. The integration of HRV measures into neurophysiological research also facilitates more accessible and cost-effective monitoring, expanding the potential for real-world applications.

The findings of this study hold significant implications for therapeutic interventions targeting cognitive deficits and autonomic dysregulation. Enhanced delta and beta band activity in regions such as the hippocampus and prefrontal cortex suggests potential benefits for individuals with memory impairments associated with neurodegenerative conditions, including Alzheimer disease and mild cognitive impairment (San-Martin et al., 2021). The observed increases in HRV indices further highlight the potential of auditory stimulation as a non-invasive intervention for managing stress-related disorders, such as generalized anxiety disorder and post-traumatic stress disorder (Poerio et al., 2018; Hooper, 2023).

Autonomic dysregulation, characterized by reduced HRV and diminished parasympathetic activity, is a hallmark of these conditions (van Ravenswaaij-Arts et al., 1993). By promoting parasympathetic activation and restoring autonomic balance, stimulation may complement existing therapeutic modalities (Kim et al., 2022; Tosti et al., 2024). Additionally, the identification of HRV as a biomarker for responsiveness supports the development of personalized interventions tailored to individual physiological profiles, optimizing therapeutic efficacy (Kim et al., 2022). The non-invasive nature of auditory stimulation, combined with its ease of application, makes it an attractive option for addressing cognitive and emotional impairments across diverse populations (Bremner et al., 2020). These findings underscore the importance of further exploring its clinical potential through rigorous trials and multidisciplinary collaborations (Tosti et al., 2024).

This study has several limitations that should be acknowledged to properly contextualize the findings. First, the absence of a control group limits causal inference, as improvements in cognitive performance and EEG measures may partially reflect practice effects rather than specific ASMR stimulation. Future studies should incorporate placebo or sham auditory conditions to isolate ASMR-specific contributions. Second, the small, homogeneous sample of Korean participants further restricts generalizability. Broader recruitment including diverse demographic and cultural backgrounds, and consideration of factors such as education and socioeconomic status, would enhance external validity. Third, biological variables such as the menstrual cycle in female participants were not controlled and could have influenced autonomic or neurophysiological responses. Additionally, participants were instructed to lie supine with eyes closed to minimize motion and ocular artifacts; however, this posture may differ from the typical ASMR experience and could affect EEG patterns. Fourth, this study employed a single auditory stimulus dominated by natural sounds (e.g., wind, birds). Although these sounds are known to promote relaxation and increase HRV, standardized criteria or objective biomarkers for ASMR remain undeveloped. Importantly, we did not perform subjective assessments to confirm whether participants experienced ASMR-specific sensations such as tingling. This methodological limitation makes it difficult to differentiate between general relaxation responses induced by nature sounds (auditory stimuli → general relaxation → EEG/ECG changes) and true ASMR-related physiological reactions (auditory stimuli → ASMR → EEG/ECG changes). Consequently, the observed effects may partly reflect general restorative autonomic processes rather than solely ASMR-specific mechanisms. Finally, our findings demonstrate the feasibility of HRV-based unsupervised clustering for identifying distinct physiological response patterns to auditory stimuli. While cross-validation supported internal consistency, the lack of subjective data limits interpretability. Future research should combine validated self-report instruments (e.g., ASMR-15, AEQ) with physiological and neural markers to establish a more robust ground truth for ASMR classification. Furthermore, real-time monitoring of HRV and multimodal imaging (e.g., fMRI) could better capture dynamic cortical–subcortical interactions. Longitudinal and randomized controlled trials in diverse populations will be essential to confirm the persistence and therapeutic potential of ASMR-induced neurocognitive changes.

This study highlights the potential of auditory stimulation as a non-invasive tool for enhancing cognitive and emotional well-being. The observed improvements in executive function, memory recall, and neural connectivity underscore its promise for addressing cognitive deficits and autonomic dysregulation. The identification of HRV indices as biomarkers for responsiveness advances the development of personalized interventions, allowing for tailored therapeutic approaches. Future research should address the challenges identified in this study, while expanding the evidence base for mechanisms and clinical applications. With rigorous validation and clinical evaluation, auditory stimulation may emerge as a valuable approach for promoting mental health and improving quality of life.

## 5 Conclusion

This study investigated the cognitive, neurophysiological, and autonomic effects of auditory stimulation using ASMR-related natural sounds, providing both theoretical and translational implications. Participants showed significant improvements in executive function and memory recall, demonstrated by reduced completion times on the Trail Making Test Part B and enhanced delayed recall performance. Neurophysiological analyses revealed increased power spectral density in the Fz and Pz regions and strengthened functional connectivity in the T8 and F8 channels, suggesting activation of neural networks associated with cognitive control and emotional regulation. HRV indices, including pNN50 and total power, emerged as sensitive indicators reflecting auditory-induced autonomic modulation and cognitive enhancement. Rather than focusing solely on responder classification, this study highlights the feasibility of HRV as a practical, non-invasive biomarker for real-time monitoring of auditory stimulation–related neurocognitive changes. These findings advance understanding of the dynamic coupling between central and autonomic systems and support the development of wearable-based assessment frameworks for cognitive and emotional monitoring. Future research should address current limitations by including more diverse populations and multimodal sensory stimuli, and by integrating advanced neuroimaging techniques to further elucidate the underlying mechanisms. With continued methodological refinement and validation, HRV-guided auditory stimulation paradigms may contribute to innovative, personalized strategies for cognitive enhancement and mental wellbeing.

## Data Availability

The raw data supporting the conclusions of this article will be made available by the authors, without undue reservation.
