# Supplementary material for: Neurophysiological and cognitive enhancements in autonomous sensory meridian response identified using heart rate variability and electroencephalography connectivity
Source: Front Psychol. 2025 Nov 24;16:1652185. doi: 10.3389/fpsyg.2025.1652185 (PMC12682686; doi:10.3389/fpsyg.2025.1652185)
Supplement: Supplementary file 1 [file Table_1.docx]

**Supplementary Table S1** Statistically significant power spectral density (PSD) changes in post-ASMR compared to pre-ASMR.

| **Frequency** | **Channel** | **Pre-ASMR** | **Post-ASMR** | ***P* value** |
| --- | --- | --- | --- | --- |
| Delta | T8 | 0.438 (0.261-0.590) | 0.529 (0.389-0.637) | 0.007 |
| Delta | Pz | 0.317 (0.158-0.401) | 0.417 (0.283-0.558) | 0.014 |
| Theta | F7 | 0.120 (0.104-0.144) | 0.114 (0.081-0.131) | 0.048 |
| Beta | Fp2 | 0.136 (0.115-0.180) | 0.158 (0.132-0.182) | 0.024 |
| Beta | Fz | 0.117 (0.048-0.174) | 0.147 (0.093-0.183) | 0.030 |
| Gamma | Fz | 0.136 (0.101-0.161) | 0.154 (0.137-0.180) | 0.015 |

Values are medians with interquartile range printed between parentheses.

**Supplementary Figure S1** Functional connectivity (FC) in terms of coherence. The plots show adjacent matrices of the coherence between 19 pairs of scalp electroencephalography electrodes for each frequency band in pre-ASMR (upper panel), post-ASMR (middle panel) and statistical significance (lower panel).


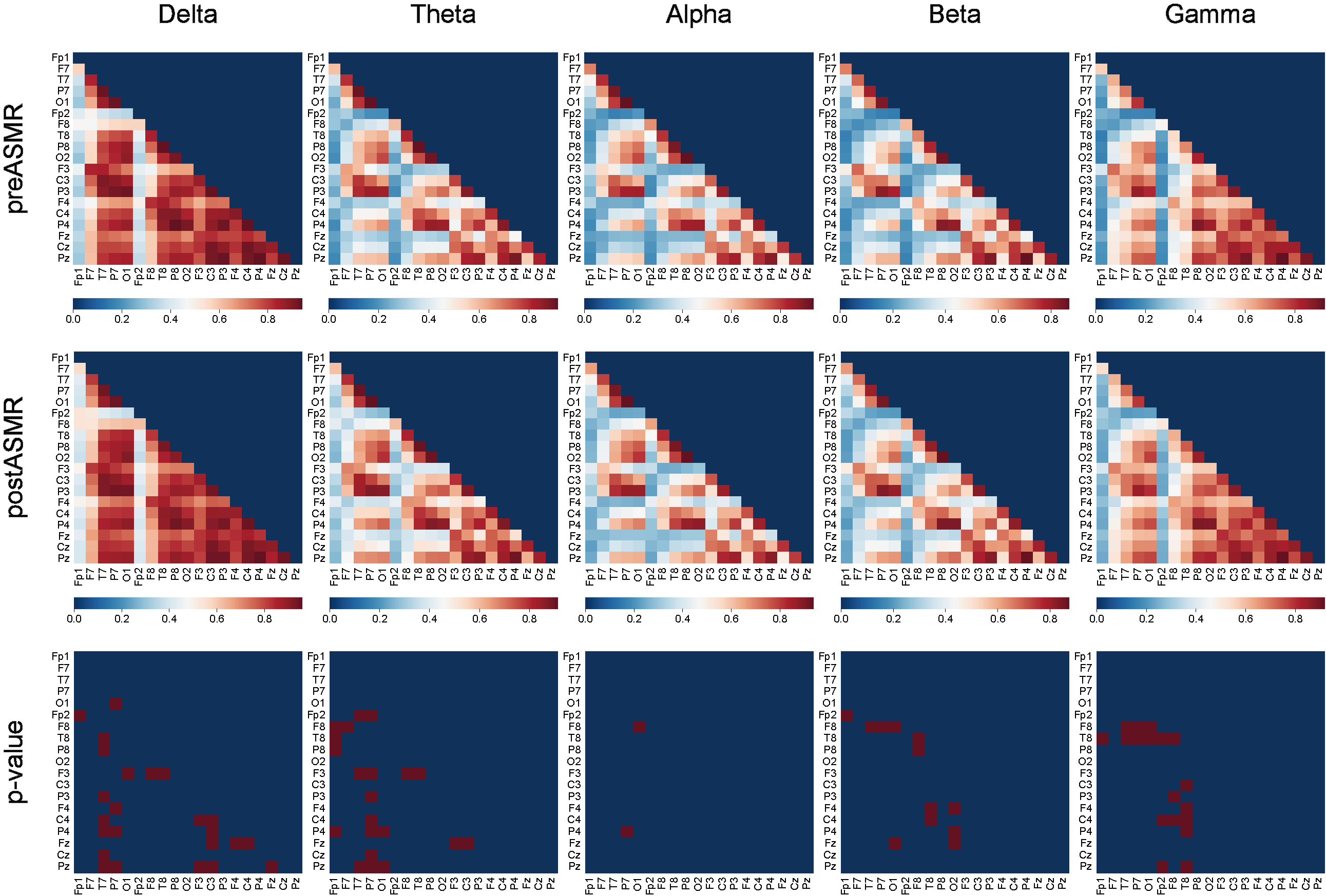


**Supplementary Table S2** Statistically significant functional connectivity (FC) changes in post-ASMR compared to pre-ASMR.

| **Coherence** | **Pre-ASMR** | **Post-ASMR** | ***P* value** |
| --- | --- | --- | --- |
| **Delta** | | | |
| O1-P7 | 0.949 (0.916-0.973) | 0.960 (0.932-0.982) | 0.030 |
| Fp2-Fp1 | 0.419 (0.297-0.532) | 0.550 (0.341-0.680) | 0.033 |
| T8-T7 | 0.757 (0.643-0.884) | 0.838 (0.727-0.930) | 0.014 |
| P8-T7 | 0.827 (0.735-0.888) | 0.866 (0.778-0.937) | 0.023 |
| F3-O1 | 0.730 (0.642-0.819) | 0.766 (0.692-0.874) | 0.037 |
| F3-F8 | 0.466 (0.212-0.685) | 0.598 (0.340-0.795) | 0.014 |
| F3-T8 | 0.599 (0.417-0.807) | 0.734 (0.575-0.867) | 0.015 |
| P3-T7 | 0.886 (0.865-0.938) | 0.927 (0.885-0.959) | 0.030 |
| F4-P7 | 0.671 (0.522-0.829) | 0.755 (0.622-0.884) | 0.040 |
| C4-T7 | 0.774 (0.646-0.895) | 0.851 (0.726-0.936) | 0.014 |
| C4-F3 | 0.792 (0.638-0.866) | 0.811 (0.659-0.904) | 0.040 |
| C4-C3 | 0.891 (0.802-0.935) | 0.906 (0.834-0.966) | 0.028 |
| P4-T7 | 0.806 (0.744-0.899) | 0.863 (0.807-0.944) | 0.019 |
| P4-P7 | 0.874 (0.778-0.935) | 0.896 (0.843-0.961) | 0.048 |
| P4-C3 | 0.884 (0.847-0.935) | 0.905 (0.870-0.957) | 0.033 |
| Fz-C3 | 0.851 (0.742-0.916) | 0.895 (0.822-0.939) | 0.030 |
| Fz-F4 | 0.805 (0.736-0.914) | 0.904 (0.747-0.955) | 0.037 |
| Fz-C4 | 0.838 (0.780-0.888) | 0.895 (0.825-0.932) | 0.028 |
| Cz-T7 | 0.766 (0.702-0.878) | 0.852 (0.757-0.922) | 0.048 |
| Pz-T7 | 0.808 (0.734-0.912) | 0.882 (0.820-0.939) | 0.028 |
| Pz-P7 | 0.841 (0.778-0.935) | 0.880 (0.850-0.952) | 0.048 |
| Pz-F3 | 0.780 (0.705-0.866) | 0.835 (0.743-0.908) | 0.040 |
| Pz-C3 | 0.943 (0.890-0.961) | 0.952 (0.923-0.972) | 0.030 |
| Pz-Fz | 0.819 (0.729-0.876) | 0.880 (0.822-0.905) | 0.023 |
| **Theta** | | | |
| Fp2-T7 | 0.236 (0.143-0.286) | 0.267 (0.245-0.330) | 0.021 |
| Fp2-P7 | 0.187 (0.117-0.253) | 0.225 (0.184-0.285) | 0.017 |
| F8-Fp1 | 0.278 (0.161-0.333) | 0.363 (0.277-0.490) | 0.048 |
| F8-F7 | 0.231 (0.143-0.324) | 0.307 (0.206-0.401) | 0.019 |
| T8-Fp1 | 0.209 (0.150-0.234) | 0.253 (0.236-0.342) | 0.015 |
| P8-Fp1 | 0.175 (0.155-0.215) | 0.230 (0.159-0.277) | 0.048 |
| F3-T7 | 0.621 (0.548-0.668) | 0.649 (0.567-0.732) | 0.028 |
| F3-P7 | 0.470 (0.367-0.548) | 0.497 (0.409-0.649) | 0.044 |
| F3-F8 | 0.203 (0.159-0.302) | 0.277 (0.195-0.369) | 0.017 |
| F3-T8 | 0.266 (0.199-0.414) | 0.333 (0.228-0.526) | 0.028 |
| P3-P7 | 0.843 (0.822-0.867) | 0.866 (0.833-0.897) | 0.030 |
| C4-P7 | 0.484 (0.348-0.572) | 0.516 (0.439-0.618) | 0.030 |
| P4-Fp1 | 0.162 (0.131-0.206) | 0.216 (0.170-0.269) | 0.021 |
| P4-P7 | 0.594 (0.550-0.705) | 0.669 (0.604-0.756) | 0.017 |
| P4-O1 | 0.661 (0.584-0.745) | 0.703 (0.624-0.794) | 0.033 |
| Fz-F3 | 0.735 (0.596-0.825) | 0.749 (0.703-0.831) | 0.025 |
| Fz-C3 | 0.599 (0.441-0.680) | 0.622 (0.535-0.774) | 0.044 |
| Cz-P7 | 0.410 (0.280-0.519) | 0.445 (0.350-0.576) | 0.021 |
| Pz-T7 | 0.596 (0.490-0.685) | 0.640 (0.535-0.728) | 0.044 |
| Pz-P7 | 0.591 (0.524-0.684) | 0.660 (0.578-0.755) | 0.011 |
| Pz-O1 | 0.613 (0.542-0.701) | 0.689 (0.616-0.747) | 0.037 |
| **Alpha** | | | |
| F8-O1 | 0.407 (0.333-0.506) | 0.454 (0.365-0.538) | 0.044 |
| P4-P7 | 0.608 (0.511-0.693) | 0.642 (0.596-0.703) | 0.040 |
| **Beta** | | | |
| Fp2-Fp1 | 0.229 (0.183-0.300) | 0.262 (0.220-0.377) | 0.044 |
| F8-T7 | 0.186 (0.137-0.257) | 0.222 (0.186-0.308) | 0.012 |
| F8-P7 | 0.253 (0.173-0.308) | 0.269 (0.211-0.357) | 0.007 |
| F8-O1 | 0.265 (0.220-0.306) | 0.300 (0.225-0.376) | 0.044 |
| T8-F8 | 0.710 (0.532-0.737) | 0.722 (0.612-0.788) | 0.011 |
| P8-F8 | 0.527 (0.472-0.597) | 0.576 (0.513-0.641) | 0.023 |
| F4-T8 | 0.478 (0.339-0.570) | 0.538 (0.417-0.575) | 0.040 |
| F4-O2 | 0.294 (0.234-0.385) | 0.329 (0.301-0.410) | 0.037 |
| C4-T8 | 0.645 (0.499-0.724) | 0.714 (0.613-0.792) | 0.023 |
| P4-O2 | 0.822 (0.791-0.862) | 0.841 (0.794-0.882) | 0.044 |
| Fz-O1 | 0.202 (0.149-0.294) | 0.234 (0.196-0.386) | 0.014 |
| Fz-O2 | 0.217 (0.173-0.337) | 0.260 (0.230-0.375) | 0.025 |
| **Gamma** |  |  |  |
| F8-T7 | 0.245 (0.193-0.344) | 0.299 (0.223-0.433) | 0.008 |
| F8-P7 | 0.301 (0.264-0.424) | 0.373 (0.281-0.489) | 0.005 |
| F8-O1 | 0.353 (0.299-0.440) | 0.443 (0.332-0.514) | 0.006 |
| T8-Fp1 | 0.143 (0.116-0.173) | 0.186 (0.149-0.248) | 0.001 |
| T8-T7 | 0.311 (0.251-0.513) | 0.501 (0.271-0.607) | 0.033 |
| T8-P7 | 0.426 (0.291-0.611) | 0.575 (0.377-0.680) | 0.019 |
| T8-O1 | 0.422 (0.312-0.608) | 0.596 (0.395-0.708) | 0.019 |
| T8-Fp2 | 0.217 (0.168-0.261) | 0.261 (0.212-0.342) | 0.033 |
| T8-F8 | 0.603 (0.418-0.667) | 0.626 (0.490-0.760) | 0.011 |
| C3-T8 | 0.465 (0.341-0.616) | 0.578 (0.465-0.693) | 0.030 |
| P3-F8 | 0.361 (0.311-0.505) | 0.440 (0.342-0.520) | 0.025 |
| F4-T8 | 0.533 (0.393-0.673) | 0.655 (0.437-0.767) | 0.044 |
| C4-Fp2 | 0.202 (0.165-0.275) | 0.244 (0.211-0.324) | 0.030 |
| C4-F8 | 0.598 (0.495-0.666) | 0.624 (0.423-0.748) | 0.033 |
| C4-T8 | 0.605 (0.445-0.706) | 0.737 (0.474-0.857) | 0.015 |
| P4-T8 | 0.626 (0.471-0.757) | 0.752 (0.538-0.823) | 0.021 |
| Pz-Fp2 | 0.159 (0.135-0.202) | 0.192 (0.152-0.275) | 0.033 |
| Pz-T8 | 0.576 (0.453-0.728) | 0.687 (0.496-0.782) | 0.021 |

Values are medians with interquartile range printed between parentheses.

**Supplementary Table S3** Statistically significant functional connectivity (FC) changes in post-ASMR compared to pre-ASMR. Significantly different features (Mann-Whitney U test, *P* < 0.05) between pre-ASMR and post-ASMR are reported in bold.

| **Features (unit)** | **Pre-ASMR** | **Post-ASMR** | ***P* value** |
| --- | --- | --- | --- |
| MeanRR (ms) | 901.478 (876.684-915.292) | 907.629 (880.420-934.867) | 0.911 |
| SDNN (ms) | 108.295 (49.342-139.409) | 58.178 (45.198-81.987) | 0.079 |
| STD HR (1/min) | 22.076 (3.559-29.133) | 4.115 (3.267-10.422) | 0.204 |
| Mean HR (1/min) | 66.557 (65.553-68.441) | 66.107 (64.183-68.150) | 0.911 |
| Min HR (1/min) | 61.277 (60.340-62.324) | 61.162 (60.480-63.683) | 0.296 |
| Max HR (1/min) | 104.156 (69.507-164.941) | 70.303 (69.426-94.810) | 0.296 |
| RMSSD (ms) | 111.069 (66.664-125.397) | 82.415 (58.234-104.304) | 0.204 |
| **NN50 (count)** | **33.000 (22.000-53.750)** | **23.000 (9.750-35.250)** | **0.029** |
| pNN50 (%) | 40.538 (19.345-54.110) | 38.040 (13.418-58.012) | 0.526 |
| RRtri (a.u.) | 10.322 (8.563-13.333) | 9.369 (7.028-12.300) | 0.057 |
| TINN (ms) | 520.500 (183.250-651.500) | 198.500 (174.250-441.250) | 0.052 |
| VLF (Hz) | 0.040 (0.037-0.040) | 0.040 (0.033-0.040) | 0.182 |
| LF (Hz) | 0.087 (0.060-0.115) | 0.092 (0.058-0.116) | 0.904 |
| HF (Hz) | 0.232 (0.202-0.322) | 0.277 (0.234-0.335) | 0.490 |
| VLF (ms2) | 65.798 (39.038-366.073) | 56.980 (13.759-213.193) | 0.550 |
| LF (ms2) | 1608.802 (642.067-3047.599) | 541.257 (299.627-1316.952) | 0.145 |
| HF (ms2) | 3296.924 (1181.994-8241.978) | 1428.137 (769.493-2782.569) | 0.550 |
| VLF (log) | 4.184 (3.665-5.892) | 4.043 (2.620-5.361) | 0.279 |
| LF (log) | 7.380 (6.452-8.022) | 6.293 (5.697-7.181) | 0.067 |
| HF (log) | 8.069 (7.071-9.004) | 7.263 (6.642-7.930) | 0.313 |
| VLF (%) | 2.043 (1.370-2.831) | 2.954 (0.892-4.443) | 0.351 |
| LF (%) | 29.427 (24.859-50.059) | 31.335 (19.113-43.578) | 0.823 |
| HF (%) | 68.342 (47.892-72.470) | 65.435 (51.215-77.285) | 1.000 |
| LF (n.u.) | 29.869 (25.475-51.144) | 32.246 (19.549-46.499) | 0.911 |
| HF(n.u.) | 69.741 (48.826-74.265) | 67.329 (53.127-79.941) | 0.940 |
| Total power (ms2) | 5049.573 (1962.482-11393.285) | 2168.394 (1323.447-3549.747) | 0.391 |
| LF/HF (a.u.) | 0.429 (0.343-1.049) | 0.479 (0.245-0.876) | 0.911 |
| SD1 (ms) | 79.048 (47.455-89.250) | 58.757 (41.550-74.367) | 0.218 |
| SD2 (ms) | 130.478 (51.306-177.896) | 59.644 (42.624-91.071) | 0.067 |
| **SD2/SD1 (a.u.)** | **1.564 (1.150-1.997)** | **1.079 (0.968-1.456)** | **0.003** |

Values are medians with interquartile range printed between parentheses. a.u.=arbitrary unit; n.u.=normalized units.

**Supplementary Table S4** Mann-Whitney U test results for the cluster using heart rate variability features. Significantly different features (Mann-Whitney U test, *P* < 0.05) between pre-ASMR and post-ASMR are reported in **bold**.

| **Features** | **K-means Clustering** | | **Mann-Whitney U Test** | | | |  |
| --- | --- | --- | --- | --- | --- | --- | --- |
|  | **Cluster 1 (*N* = 14)** | **Cluster 2 (*N* = 4)** | | **U** | ***Z*** | ***P* value** | |
| **Delayed recall** | **1.071 ± 1.439** | **-0.500 ± 0.577** | | **6.000** | **-2.474** | **0.018** | |
| Trail Making Test | -1.804 ± 3.124 | -1.490 ± 2.397 | | 26.000 | -0.212 | 0.878 | |
| Delta T8 | 0.080 ± 0.115 | 0.101 ± 0.127 | | 25.000 | -0.319 | 0.798 | |
| Delta Pz | 0.118 ± 0.176 | 0.115 ± 0.076 | | 28.000 | 0.000 | 1.000 | |
| Theta F7 | -0.022 ± 0.048 | -0.032 ± 0.062 | | 23.000 | -0.531 | 0.645 | |
| Beta Fp2 | 0.036 ± 0.079 | 0.017 ± 0.042 | | 24.000 | -0.425 | 0.721 | |
| Beta Fz | 0.041 ± 0.075 | 0.039 ± 0.061 | | 27.000 | -0.106 | 0.959 | |
| Gamma Fz | 0.022 ± 0.036 | 0.016 ± 0.025 | | 25.000 | -0.319 | 0.798 | |
| Delta O1-P7 | 0.013 ± 0.032 | 0.001 ± 0.020 | | 22.000 | -0.637 | 0.574 | |
| Delta Fp2-Fp1 | 0.161 ± 0.192 | 0.123 ± 0.321 | | 21.000 | -0.743 | 0.505 | |
| Delta T8-T7 | 0.081 ± 0.153 | 0.047 ± 0.126 | | 24.000 | -0.425 | 0.721 | |
| Delta P8-T7 | 0.059 ± 0.117 | 0.024 ± 0.093 | | 23.000 | -0.531 | 0.645 | |
| Delta F3-O1 | 0.081 ± 0.188 | 0.039 ± 0.111 | | 26.000 | -0.212 | 0.878 | |
| Delta F3-F8 | 0.119 ± 0.261 | 0.134 ± 0.264 | | 28.000 | 0.000 | 1.000 | |
| Delta F3-T8 | 0.105 ± 0.226 | 0.102 ± 0.172 | | 27.000 | -0.106 | 0.959 | |
| Delta P3-T7 | 0.034 ± 0.055 | 0.025 ± 0.059 | | 27.000 | -0.106 | 0.959 | |
| Delta F4-P7 | 0.063 ± 0.202 | 0.077 ± 0.194 | | 20.000 | -0.850 | 0.442 | |
| Delta C4-T7 | 0.061 ± 0.134 | 0.054 ± 0.150 | | 27.000 | -0.106 | 0.959 | |
| Delta C4-F3 | 0.048 ± 0.154 | 0.079 ± 0.127 | | 22.000 | -0.637 | 0.574 | |
| Delta C4-C3 | 0.017 ± 0.099 | 0.032 ± 0.095 | | 27.000 | -0.106 | 0.959 | |
| Delta P4-T7 | 0.061 ± 0.105 | 0.014 ± 0.099 | | 23.000 | -0.531 | 0.645 | |
| Delta P4-P7 | 0.054 ± 0.093 | 0.014 ± 0.068 | | 27.000 | -0.106 | 0.959 | |
| Delta P4-C3 | 0.031 ± 0.079 | -0.002 ± 0.057 | | 18.000 | -1.062 | 0.327 | |
| Delta Fz-C3 | 0.032 ± 0.141 | 0.072 ± 0.109 | | 19.000 | -0.956 | 0.382 | |
| Delta Fz-F4 | 0.037 ± 0.125 | 0.084 ± 0.112 | | 24.000 | -0.425 | 0.721 | |
| Delta Fz-C4 | 0.028 ± 0.130 | 0.065 ± 0.088 | | 21.000 | -0.743 | 0.505 | |
| Delta Cz-T7 | 0.064 ± 0.102 | 0.055 ± 0.146 | | 20.000 | -0.850 | 0.442 | |
| Delta Pz-T7 | 0.077 ± 0.118 | 0.026 ± 0.092 | | 24.000 | -0.425 | 0.721 | |
| Delta Pz-P7 | 0.067 ± 0.120 | 0.024 ± 0.060 | | 27.000 | -0.106 | 0.959 | |
| Delta Pz-F3 | 0.057 ± 0.093 | 0.042 ± 0.089 | | 26.000 | -0.212 | 0.878 | |
| Delta Pz-C3 | 0.033 ± 0.086 | 0.005 ± 0.035 | | 16.000 | -1.274 | 0.233 | |
| Delta Pz-Fz | 0.061 ± 0.145 | 0.037 ± 0.088 | | 25.000 | -0.319 | 0.798 | |
| Theta Fp2-T7 | 0.160 ± 0.208 | -0.026 ± 0.133 | | 11.000 | -1.805 | 0.079 | |
| Theta Fp2-P7 | 0.139 ± 0.218 | -0.006 ± 0.111 | | 14.000 | -1.487 | 0.158 | |
| Theta F8-Fp1 | 0.191 ± 0.290 | -0.011 ± 0.074 | | 11.000 | -1.805 | 0.079 | |
| Theta F8-F7 | 0.141 ± 0.218 | -0.111 ± 0.235 | | 14.000 | -1.487 | 0.158 | |
| Theta T8-Fp1 | 0.134 ± 0.253 | 0.039 ± 0.022 | | 14.000 | -1.487 | 0.158 | |
| Theta P8-Fp1 | 0.104 ± 0.248 | 0.014 ± 0.058 | | 18.000 | -1.062 | 0.327 | |
| Theta F3-T7 | 0.102 ± 0.166 | -0.020 ± 0.172 | | 19.000 | -0.956 | 0.382 | |
| Theta F3-P7 | 0.108 ± 0.203 | -0.010 ± 0.146 | | 19.000 | -0.956 | 0.382 | |
| Theta F3-F8 | 0.156 ± 0.207 | -0.052 ± 0.148 | | 11.000 | -1.805 | 0.079 | |
| Theta F3-T8 | 0.135 ± 0.219 | 0.002 ± 0.228 | | 27.000 | -0.106 | 0.959 | |
| **Theta P3-P7** | **0.049 ± 0.071** | **-0.015 ± 0.032** | | **7.000** | **-2.230** | **0.025** | |
| Theta C4-P7 | 0.097 ± 0.216 | 0.010 ± 0.108 | | 23.000 | -0.531 | 0.645 | |
| Theta P4-Fp1 | 0.134 ± 0.241 | 0.013 ± 0.021 | | 10.000 | -1.912 | 0.061 | |
| Theta P4-P7 | 0.086 ± 0.171 | -0.008 ± 0.088 | | 17.000 | -1.168 | 0.277 | |
| Theta P4-O1 | 0.088 ± 0.174 | -0.015 ± 0.090 | | 15.000 | -1.381 | 0.192 | |
| Theta Fz-F3 | 0.062 ± 0.107 | 0.063 ± 0.122 | | 27.000 | -0.106 | 0.959 | |
| Theta Fz-C3 | 0.088 ± 0.130 | 0.051 ± 0.116 | | 25.000 | -0.319 | 0.798 | |
| Theta Cz-P7 | 0.103 ± 0.221 | 0.009 ± 0.096 | | 21.000 | -0.743 | 0.505 | |
| Theta Pz-T7 | 0.117 ± 0.197 | -0.046 ± 0.102 | | 11.000 | -1.805 | 0.079 | |
| **Theta Pz-P7** | **0.120 ± 0.198** | **-0.046 ± 0.060** | | **6.000** | **-2.336** | **0.018** | |
| **Theta Pz-O1** | **0.121 ± 0.209** | **-0.066 ± 0.079** | | **5.000** | **-2.443** | **0.012** | |
| Alpha F8-O1 | 0.118 ± 0.146 | -0.103 ± 0.206 | | 10.000 | -1.912 | 0.061 | |
| Alpha P4-P7 | 0.096 ± 0.164 | -0.043 ± 0.092 | | 12.000 | -1.699 | 0.101 | |
| Beta Fp2-Fp1 | 0.094 ± 0.233 | 0.092 ± 0.066 | | 22.000 | -0.637 | 0.574 | |
| **Beta F8-T7** | **0.110 ± 0.213** | **-0.029 ± 0.060** | | **8.000** | **-2.124** | **0.035** | |
| Beta F8-P7 | 0.099 ± 0.217 | -0.008 ± 0.052 | | 11.000 | -1.805 | 0.079 | |
| Beta F8-O1 | 0.090 ± 0.211 | 0.001 ± 0.093 | | 18.000 | -1.062 | 0.327 | |
| Beta T8-F8 | 0.086 ± 0.122 | 0.024 ± 0.053 | | 21.000 | -0.743 | 0.505 | |
| Beta P8-F8 | 0.063 ± 0.110 | -0.002 ± 0.038 | | 15.000 | -1.381 | 0.192 | |
| Beta F4-T8 | 0.106 ± 0.177 | -0.007 ± 0.079 | | 15.000 | -1.381 | 0.192 | |
| Beta F4-O2 | 0.082 ± 0.194 | 0.014 ± 0.099 | | 22.000 | -0.637 | 0.574 | |
| Beta C4-T8 | 0.125 ± 0.179 | -0.054 ± 0.121 | | 9.000 | -2.018 | 0.046 | |
| Beta P4-O2 | 0.046 ± 0.071 | -0.011 ± 0.062 | | 18.000 | -1.062 | 0.327 | |
| Beta Fz-O1 | 0.093 ± 0.233 | 0.015 ± 0.029 | | 15.000 | -1.381 | 0.192 | |
| Beta Fz-O2 | 0.091 ± 0.219 | 0.015 ± 0.093 | | 22.000 | -0.637 | 0.574 | |
| Gamma F8-T7 | 0.130 ± 0.183 | 0.010 ± 0.056 | | 11.000 | -1.805 | 0.079 | |
| Gamma F8-P7 | 0.103 ± 0.189 | 0.016 ± 0.058 | | 21.000 | -0.743 | 0.505 | |
| Gamma F8-O1 | 0.105 ± 0.174 | 0.021 ± 0.095 | | 16.000 | -1.274 | 0.233 | |
| Gamma T8-Fp1 | 0.087 ± 0.233 | 0.074 ± 0.087 | | 22.000 | -0.637 | 0.574 | |
| **Gamma T8-T7** | **0.126 ± 0.209** | **-0.034 ± 0.043** | | **8.000** | **-2.124** | **0.035** | |
| Gamma T8-P7 | 0.119 ± 0.213 | -0.014 ± 0.061 | | 14.000 | -1.487 | 0.158 | |
| Gamma T8-O1 | 0.125 ± 0.211 | -0.016 ± 0.066 | | 12.000 | -1.699 | 0.101 | |
| Gamma T8-Fp2 | 0.092 ± 0.220 | 0.012 ± 0.087 | | 19.000 | -0.956 | 0.382 | |
| Gamma T8-F8 | 0.091 ± 0.136 | 0.028 ± 0.100 | | 18.000 | -1.062 | 0.327 | |
| Gamma C3-T8 | 0.118 ± 0.192 | -0.010 ± 0.053 | | 14.000 | -1.487 | 0.158 | |
| Gamma P3-F8 | 0.080 ± 0.138 | 0.004 ± 0.078 | | 17.000 | -1.168 | 0.277 | |
| Gamma F4-T8 | 0.114 ± 0.171 | 0.004 ± 0.134 | | 18.000 | -1.062 | 0.327 | |
| Gamma C4-Fp2 | 0.092 ± 0.212 | 0.002 ± 0.096 | | 19.000 | -0.956 | 0.382 | |
| Gamma C4-F8 | 0.070 ± 0.113 | 0.085 ± 0.127 | | 27.000 | -0.106 | 0.959 | |
| Gamma C4-T8 | 0.125 ± 0.169 | 0.026 ± 0.104 | | 18.000 | -1.062 | 0.327 | |
| Gamma P4-T8 | 0.110 ± 0.176 | -0.011 ± 0.096 | | 14.000 | -1.487 | 0.158 | |
| Gamma Pz-Fp2 | 0.080 ± 0.226 | 0.005 ± 0.062 | | 21.000 | -0.743 | 0.505 | |
| Gamma Pz-T8 | 0.108 ± 0.178 | -0.007 ± 0.084 | | 16.000 | -1.274 | 0.233 | |

Values are indicated mean ± standard deviation of difference (post-ASMR – pre-ASMR).
